# Females Are More Aggressive Than Males towards Same- and Opposite-Sex Intruders in the Blue Tit (*Cyanistes caeruleus*)

**DOI:** 10.3390/ani13040585

**Published:** 2023-02-07

**Authors:** Gust Boiten, Robin van Iersel, Rianne Pinxten, Marcel Eens

**Affiliations:** 1 Behavioural Ecology and Ecophysiology Group, Department of Biology, University of Antwerp, 2610 Antwerp, Belgium; 2 Research Group Didactica, Antwerp School of Education, University of Antwerp, 2610 Antwerp, Belgium

**Keywords:** aggression, conspecific competition, sex-specific aggression, intersexual aggression, intrasexual aggression, song, simulated territorial intrusion

## Abstract

**Simple Summary:**

Animals use aggressive behaviour to gain access to resources such as food and mates, and protect their offspring. Individuals vary in the way they express aggression during a conflict. However, our understanding of female aggression is limited. In this study, we investigated male and female aggression towards opponents of the same and opposite sex in the blue tit (*Cyanistes caeruleus*). Aggressive encounters were simulated during the egg-laying phase of the female using blue tit taxidermy mounts. For each sex, we identified behaviours used during aggressive encounters. We compared the intensity of same- and opposite-sex aggression and examined if individual aggression was consistent in both situations. Finally, we compared the intensity of aggression between males and females. Our results show that females are the more aggressive sex, even though both sexes showed similar behaviours during conflicts. Female and male aggression was mostly unaffected by the sex of the intruder, although males sang more from a distance when encountering a male intruder. We conclude that females might have evolved to be more aggressive as they have to compete for nesting sites, and have invested more resources into reproduction.

**Abstract:**

During the breeding season, aggression is expressed to gain access to resources such as territories and mates and protect offspring. Female aggressiveness has received much less attention than male aggressiveness, and few studies have examined female and male aggressiveness towards intruders of both sexes in the same species. We compared female and male aggressiveness towards same- and opposite-sex intruders during the egg-laying period in blue tits (*Cyanistes caeruleus*) using simulated territorial intrusions. For each sex, we examined the occurrence of different behavioural responses during agonistic encounters, and compared the intensity and individual consistency of intra- and inter-sexual aggression using same- and opposite-sex taxidermy mounts. Our results show that females are the more aggressive sex. Both sexes showed similar behaviours during simulated intrusions, although females were never observed singing and males never entered the nest box. In females, aggression was predominantly independent of the sex of the intruder, while males sang more from a distance during male–male encounters. The relative levels of aggression (pecking and perching on the mounts) during intra- and intersexual conflicts were consistent for females, but not for males. Females might be under stronger selection for aggressive phenotypes due to nest-hole competition and larger reproductive investments.

## 1. Introduction

During the breeding season, animals express aggressive behaviour to gain access to resources such as territories and potential partners [1,2]. Males are often considered to be the more (competitive) aggressive sex, as the intensity of individual aggression is often linked to steroid hormone levels [2]. Females also display aggression, but often in different contexts. Female aggression is mainly expressed to defend the offspring, so called maternal aggression [2,3]. Nevertheless, in many bird and fish species, female intra-and intersexual aggression has also been observed in competitive contexts (e.g., [1,2,3,4,5]), although this topic remains understudied [6,7].

Intra- and intersexual aggression can provide direct reproductive benefits for an individual, especially in species that lay eggs or raise dependent young. In birds, aggression against conspecifics can increase reproductive success by preventing nest occupation/destruction or brood parasitism [8]. Furthermore, in biparental species where polygyny and extra-pair copulations (EPCs) occur, individuals within a pair often have contrasting interests [3,4,5,9,10,11,12,13,14,15,16,17]. Males benefit from additional females settling nearby, providing opportunities for polygyny and extra-pair paternity. While a monogamous male puts all his paternal effort in a single nest, a polygynous male may divide his efforts over multiple nests and the total level of paternal care has been shown to be lower in polygynous compared to monogamous males, even if they do not divide efforts between nests [15]. Reduced paternal investment can negatively impact the survival of both primary and secondary females [15,18,19,20]. To maximize paternal investment for her offspring and ensure monogamy, females can use aggression to prevent or delay the settlement of additional females [4,13,14,18,21]. Furthermore, by preventing EPCs of her partner, a female can reduce the risk of disease. Similarly, males can ensure their paternity of the brood by acting aggressively towards male intruders [5]. In some species, these within-pair conflicts can even lead to aggression between partners [17,22,23]. Male European starlings (*Sturnus vulgaris*) and red-winged blackbirds (*Agelaius phoeniceus*) have been shown to chase away their primary partners to deflect female aggression towards secondary females, increasing their opportunities for polygyny. Previous research, using simulated territorial intrusions (either via taxidermy mounts or caged birds), has shown that contrasting interests between sexes often lead to a more intense response to same-sex conspecific intruders compared to opposite-sex intruders [3,4,9,11,12,13,17], although others did not find such sex-specific responses [11]. However, research comparing inter- and intra-sexual aggression in both sexes in temperate zone birds is still limited but see [9,11,12,13,17,24]. 

Individuals vary in their level of aggressiveness and can use different types of aggressive displays during intra- and intersexual agonistic encounters [18,25]. Aggression can consist of both signalling and physical aggression. The latter comes at a much greater risk of injury. Most species use signals (visual, chemical or auditory) to communicate their fighting ability or aggressive intent and resolve agonistic interactions, thereby reducing the risk of unnecessary injury [26]. Understanding the different strategies employed by males and females in a competitive context may provide insight into the underlying processes that drive aggression within each sex. In addition, individual aggressiveness can be consistent through time and context [3,25,27]. This within-individual consistency is known under various overlapping terms including temperament, coping style and personality [28]. The level of consistency in responses towards male and female intruders can reveal more about the relationship between inter- and intrasexual aggression, which can depend on separate or linked underlying (physiological) mechanisms [3,29,30].

Across species, cavity-nesting birds tend to be more aggressive compared to species with more flexible nesting strategies [11]. Cavity-nesting birds have a lower availability of nesting sites, resulting in a higher level of competition, with female aggression also playing an important role in the acquisition and retainment of nesting holes [1,11,31]. The blue tit (*Cyanistes caeruleus*) is a small, secondary cavity-nesting passerine and is one of the most aggressive bird species relative to its size [32], with both males and females being highly aggressive [5,18,21]. Blue tits have biparental care and a facultatively polygynous mating system [15]. In this species, sexual infidelity is common for both males and females [10,33], indicating both individuals within a pair often have contrasting interests, leading to inter-sexual conflicts such as mate-guarding [15,18,33]. During aggressive encounters, blue tits have been shown to use different strategies. A recent study by Velasco, Ferrer and Sanz [34] described three strategies used by male blue tits during male–male territorial conflicts, which can be ranked from high to low risk. However, research on strategies used by female blue tits during aggressive encounters with conspecifics is still lacking.

In this study, we therefore examined and compared female and male aggressiveness towards intruders of both sexes in the blue tit, during the egg-laying period, using simulated territorial intrusions. By quantifying aggression in individuals of both sexes within a pair towards either male or female taxidermy mounts (hereafter “decoys”), we addressed four main research questions. (1) Do male and female behavioural responses during aggressive encounters differ? (2) Does the aggressive response during a simulated intrusion depend on the sex of the decoy? While territoriality and protection of a nest site can result in both intra- and intersexual aggression, the polygynous mating system and the occurrence of EPCs in blue tits are expected to result in a more aggressive response towards a same-sex intruder [5,15,18,21]. Furthermore, we also examined whether within-pair aggression occurs [17,22,23]. (3) Is the relative level of aggression of individuals consistent when comparing their responses towards same-sex and opposite-sex intruders? Even though the aggressive response towards male and female decoys can differ, the relative level of aggression of individual birds is expected to be consistent (i.e., aggressive birds have the strongest response during both intra- and intersexual conflicts) [3]. (4) Does the intensity of aggressive responses differ between the sexes? Although, in general, males are considered to be the more aggressive sex [2], in species with a strong competition for nesting sites, such as blue tits, the level of female aggression is expected to be high as well [1,11,18,21,31,35,36].

## 2. Materials and Methods

### 2.1. Study Population and Field Methods

This study was conducted using a resident nest-box population of blue tits in the suburban area surrounding the University of Antwerp in Wilrijk, Belgium (51°09′46″ N, 4°24′13″ E, see [37]). In total, 61 nest boxes in the area were exclusively available for blue tits. Furthermore, some blue tits also nested in the nest boxes provided for a population of great tits (*Parus major*) in the same area. Data were collected during the breeding season of 2022 (March–May). Birds in the population were marked with individual combinations of plastic colour rings and a numbered aluminium ring in the preceding winter while roosting in nest boxes, making sexing and identification in the field possible. Each individual was sexed based on size, colour and behaviour [38]. Out of 29 tested pairs, 31.0% had both individuals ringed, 31.0% had only the female ringed, and 17.3% had only the male ringed, for a total of 79.3% of the pairs having at least one individual ringed. In addition, the age class (yearling or older) of the focal individuals was determined. Yearlings made up 42.1% of the male and 36% of the female individuals for which age was determined (79% and 96% for males and females, respectively). To identify new nests, nest boxes were checked for nest building regularly at the start of the breeding season (starting in the second week of March). Nests that were at the final stages of nest building were monitored daily to determine the first day of egg-laying. Throughout the egg-laying period, nests were regularly checked to detect possible laying interruptions. A laying interruption is a gap in egg-laying for a period of 1 day or more, often caused by external stressors such as cold weather (Described in [39]).

### 2.2. Simulated Territorial Intrusions

We obtained measures of intra- and intersexual aggression during the egg-laying period of the resident female. The territorial responses of both the male and female of a pair were recorded during simulated territorial intrusions (hereafter ‘trials’) using a male or female decoy. A camera (Sony HDR-XR550VE or JVC GZ-R405BE) was used to allow for an accurate quantification of aggressive behaviours. Three male and three female stuffed blue tits (decoys) were used (1 yearling, 2 older for each sex). Stuffed birds were placed in a small wire cage (15.5 × 15.5 × 17.5 cm) to protect them from physical damage. Three observers (G.B., R.I., or an observer trained by M.E.) were randomized over the trials and alternated the use of the decoys at random.

The trials were performed between day 2 and 4 of the egg-laying period, between 08:00–13:30. Female decoys were presented on day 3, while male decoys were presented on day 2 or 4. The order of the trials was alternated for the different nest boxes and trials with a male or female decoy at the same nest box coming at least a day apart. Trials in which the focal individuals did not show up, were repeated the same day or following days (day 4 or day 5–6 for female and male decoys, respectively). Before the start of each trial, a camera was set up at a distance of 5–7 m from the nest box. After the decoy was placed on top of the nest box, the observer took place behind the camera and waited for the pair to arrive. Both pair members were tested for 5 min after their first arrival. The 5 min test started when the individual entered a radius of 15 m from the nest box. When individuals were out of frame (approximately 1.5 m by 2.7 m), the observer described behaviours of interest, so that these could be quantified when analysing the videos. Behavioural data extracted from the video recordings were: the amount of time and the number of times perched on the decoy, the amount of time and the number of times perched in front of the nest-box entrance, the amount of time spent in the nest box and the number of times entering the nest box, the minimum distance to the decoy (0 m when perched on the decoy, 0.01 m when inside the nest box, estimated to 0.1 m accuracy when closer than a meter and 1 m accuracy when further away), the number of pecks on the decoy, the number of calls, the number of song phrases [40], and the number and direction of physical aggressive interactions within a pair. During the video analysis the individuals were sexed based on ring combinations and typical behavioural differences, such as only the females entering the nest box.

We recorded a total of 60 trials at 30 nest boxes. This resulted in 120 individual responses from resident birds of both sexes. A number of these individual responses (N_male_ = 12 at six nest boxes; N_female_ = 8 at four nest boxes) were discarded due to one of the following reasons: (1) insufficient recorded time, (2) individuals did not show up or (3) individuals did not enter the radius of 15 m. This led to different sample sizes for male and female birds. Per individual, data were only included if both trials with a male and female decoy succeeded, to make comparisons between trials possible. For 70% of the 30 nest boxes, the responses for both members of the pairs were available, while for one nest box data from both members of a pair were discarded. All data are available in Supplementary Data S1.

### 2.3. Statistical Methods

All statistics were performed using R 4.0.2 [41]. Principal component analyses (PCA) were applied to capture the variance of the behavioural variables in a few principal components (PCs), to capture the intercorrelation structure, and to identify possible strategies used during territorial intrusions. To this end, the ‘prcomp’ function from the ‘stats’ package was used in R. Separate PCA’s were completed for male and female birds, including the responses toward both male and female decoys in a single PCA. Initially, all recorded variables, except for the number of aggressive interactions within a pair, were included in the female and male PCA’s. However, due to a strong correlation (ρ > 0.90, *p* < 0.001) between the number of times perching in front of the nest-box entrance and the time spent in front of the entrance, the former variable was excluded from both PCA’s. Furthermore, the number of times perching on the decoy was excluded from the male PCA due to a strong correlation (ρ > 0.90, *p* < 0.001) with the time spent on the decoy. Singing was never observed in female birds; therefore, the number of song phrases was only included in the male PCA. Similarly, male birds were never observed entering the nest box; therefore, the amount of time and number of times entering the nest box were only included in the female PCA. All input variables were log transformed prior to analysis, to reduce the influence of extreme values. The minimum distance variable was reversed (−1), so a higher value indicates a closer distance and therefore a more aggressive response. The correlation matrix of these variables was validated using the Bartlett’s test in the ‘psych’ package (version 2.2.5) [42] in R (Male: χ^2^ = 78.40, *p* < 0.001; Female: χ^2^ = 210.83, *p* < 0.001). Sampling adequacy was determined using the Kaiser–Meyer–Olkin test from the same package (Male KMO = 0.69; Female KMO = 0.61). The principal component analysis was based on the correlation matrix and variables were centred and scaled prior to analysis. PCs with eigenvalues >1 were selected for further analysis. A cut-off value of >0.40 was used for factor loadings to identify the important variables contributing to a component.

The data we collected did not lend itself well to a mixed regression model approach, as the assumptions for the models were not met and the models consistently failed to converge. We attempted to fit a generalized linear mixed effects model, but even this more flexible approach resulted in the same issues. As a result, we employed two-sample tests instead. However, this approach had its limitations as it did not account for the different decoys and observers used, the ages of the individuals and the order of the trials. To determine the influence of these variables on the principal components and individual aggression parameters, we performed Kruskal–Wallis tests for the decoys and observers and Mann–Whitney U tests for the age and order of trials. After applying the Holm correction to control for multiple testing [43], none of these variables had significant effects (α > 0.05). 

The responses on male and female decoys were compared using paired sample tests on the individual principal component scores. The principal components of male and female individuals were analysed separately. Normally, distributed data were analysed using paired t-tests, whereas Wilcoxon signed rank tests were used for non-normal data. The relationship between an individual’s response to the decoy and the occurrence of within-pair conflicts, and the individual consistency in the level of aggression on male and female decoys were both tested using Spearman correlations. To compare the intensity of behavioural responses between the sexes, the principal components could not be used as they come from separate PCA’s. Instead, all behavioural parameters were compared separately between the sexes, using Mann–Whitney U tests.

## 3. Results

### 3.1. Identification of Female and Male Behavioural Responses

Descriptive statistics for the aggression parameters are given in Table A1, Appendix A. The PCA for females resulted in three principal components that describe a total of 78.1% of the variation (Table 1). For ease of interpretation, PC1 was multiplied by (−1) for further analysis, so a higher value indicates a higher level of aggression. Females with a high value for PC1 showed more pecks, perched more often and spent more time on the decoy and in front of the nest-box entrance, and were considered confrontational. Only three females did not perch on the decoy during one of their trials, resulting in little variation for the variable minimum distance. PC2 and PC3 described non-confrontational behaviour. PC2 described the contrast between females calling (high scores) or entering the nest box more often and for longer periods of time (low scores). High values for PC3 described both females calling and frequently entering the nest box, and females calling from a distance. None of the 26 females were observed singing during any of the trials.

The PCA for males revealed three PCs that describe a total of 79.5% of the variation (Table 2). For ease of interpretation, PC1 was multiplied by (−1) for further analysis, so a higher value indicates a higher level of aggression. Males with a high value for PC1 were confrontational: they approached the decoy more closely, displayed more pecks, and spent more time on the decoy and in front of the nest-box entrance. PC2 and PC3 described non-confrontational behaviour. High values for PC2 (mainly based on five individuals displaying song) described males displaying songs from a distance. PC3 reflected a contrast between males calling (high score) or singing (low score). None of the males entered their nest box during any of the trials. For both males and females, individuals can have high values across multiple components.

### 3.2. Response to a Same- and Opposite-Sex Intruder

**Figure 1:**
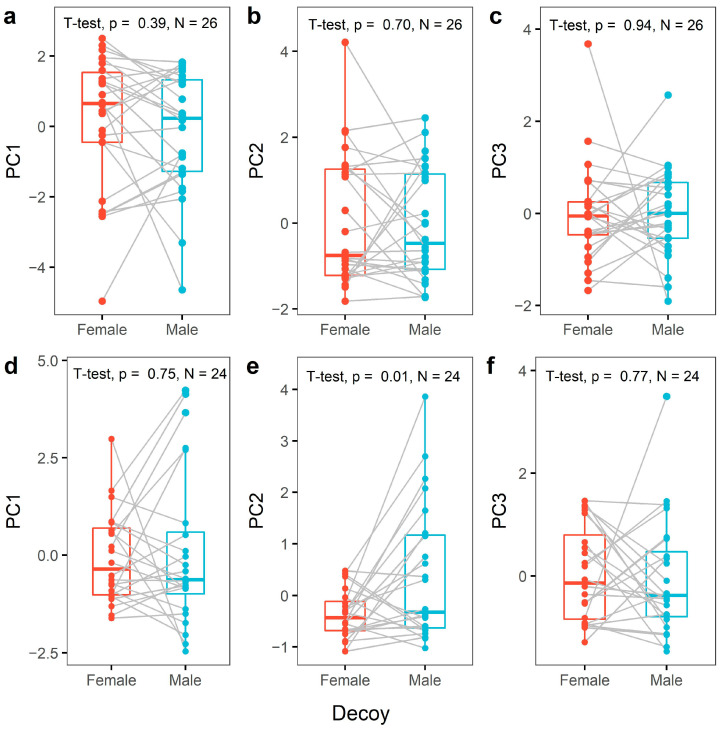
Paired boxplots showing the level of aggression during trials with a female or a male decoy. Female aggression scores based on PC1 (**a**), PC2 (**b**) and PC3 (**c**) and male aggression scores based on PC1 (**d**), PC2 (**e**) and PC3 (**f**) are shown. The grey lines show the difference in individual aggression scores between trials.

We found no significant differences in female aggressiveness between female and male decoys for any of the three PCs (*p* > 0.39 for all three PCs; Figure 1a–c). Similarly, male aggressiveness based on PC1 and PC3 did not differ between male and female decoys (*p* > 0.75). However, there was a difference in the PC2 scores between male and female intruders (t = −2.70; DF = 23; *p* = 0.013), with males displaying more songs from a distance towards a male decoy (Figure 1d–f). In total, 5 out of 24 males (20.8%) sang during a trial. Males almost exclusively sang during encounters with a male decoy. Only one out of the five singing males produced one song phrase during a trial with a female decoy, while four males produced 1–51 song phrases during trials with a male decoy. When considering the measured aggression variables separately for both sexes, we found no significant differences in responses towards male or female decoys after applying the Holm multiple testing correction [43] (Table A2, Appendix A).

### 3.3. Within-Pair Aggression

**Figure 2:**
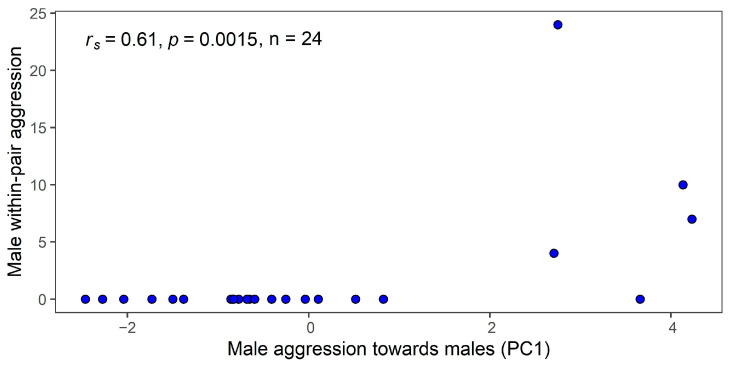
Scatterplot showing the relationship between the number of male within-pair attacks and male–male aggression based on PC1 (N_Male_ = 24). The Spearman correlation coefficient and *p*-value are shown in the top left corner.

We recorded three females and four males (from five pairs) attacking their partner. In two pairs, both the male and female displayed within-pair aggression. For females, this behaviour occurred once in the presence of a female and twice in the presence of a male decoy. Male aggressive behaviour towards their partner was only observed in the presence of a male decoy. Furthermore, males that attacked their partner were more confrontational aggressive individuals, as revealed by a highly significant correlation between the number of within-pair attacks and male aggression based on PC1 (Figure 2). For the other two PCs in males and for females, there was no significant correlation (*p* > 0.09 in all cases).

### 3.4. Consistency in Same- and Opposite-Sex Aggression

**Figure 3:**
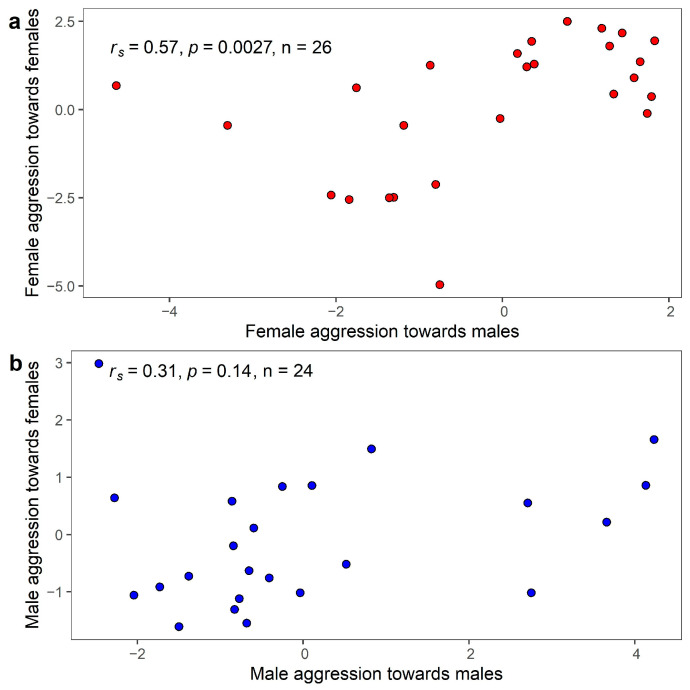
Scatterplot showing the individual consistency between intra-sexual and inter-sexual aggression based on PC1 for each sex (N_Female_ = 26; N_Male_ = 24). (**a**) Female aggression towards females versus female aggression towards males; (**b**) Male aggression towards females versus male aggression towards males. The Spearman correlation coefficient and *p*-value are presented in the top left corner.

When considering the response of each sex towards same- and opposite-sex intruders, we found a highly significant positive correlation between the level of female confrontational aggression (PC1) towards female and male intruders, but not for PC2 and PC3 (PC1: ρ = 0.57, *p* = 0.0027; PC2: ρ = 0.32, *p* = 0.11; PC3: ρ = 0.13, *p* = 0.53; Figure 3a). By contrast, in males, there was no association between the response towards male and female intruders for any of the three PCs (PC1: ρ = 0.31, *p* = 0.14; PC2: ρ = 0.15, *p* = 0.47; PC3: ρ = 0.058, *p* = 0.79; Figure 3b).

### 3.5. Sex Differences in Aggressive Behaviour

**Figure 4:**
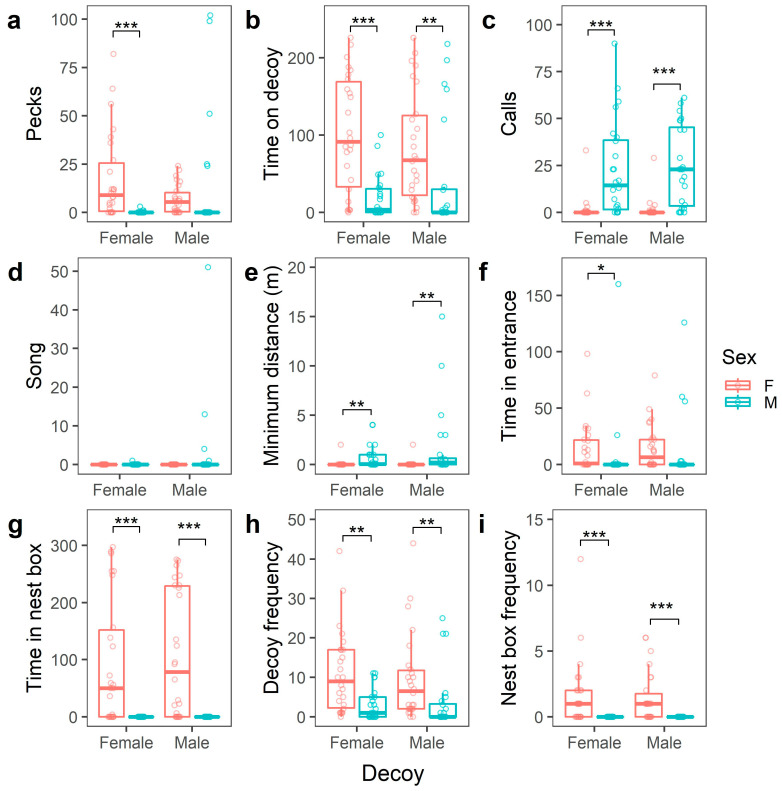
Comparison of behavioural variables between the sexes (N_Female_ = 26 in red; N_Male_ = 24 in blue) of the tested birds and the sexes of the decoys. Asterisks indicate level of statistical significance: * *p* ≤ 0.05, ** *p* ≤ 0.01, *** *p* ≤ 0.001. Behavioural variables: The number of pecks (**a**); Time spent on the decoy (**b**); The number of calls (**c**); The number of song phrases (**d**); The minimum distance to the decoy (**e**); Time spent in front of the nest-box entrance (**f**); The time spend in the nest box (**g**); The number of times perching on the decoy (**h**); The number of times entering the nest box (**i**).

In general, females showed more aggressive behaviour compared to males. Irrespective of the sex of the decoy (Table A3, Appendix A), females approached the decoys more closely, perched more frequently and spent more time on the decoys and in the nest box than males did. While almost all females perched on the decoys (88.5% perched during both trials), significantly more males stayed at a distance (29.2% perched during both trials) (Table A3, Appendix A). Females also pecked significantly more often and spent more time in the nest-box entrance, but only during trials with a female decoy (Figure 4 and Table A3, Appendix A). Finally, females called significantly less than males and were never observed singing (Table A1 and Table A3, Appendix A). Males were never observed entering the nest box during a simulated territorial intrusion (Figure 4).

## 4. Discussion

While male aggression is well-studied across taxa, research comparing intra- and intersexual aggression between both sexes within a species is limited. In this study, we assessed the aggressiveness of blue tits during the egg-laying period, in order to examine whether male and female behavioural responses varied during both intra and inter-sexual simulated territorial intrusions. 

Our principal component analyses uncovered similarities between male and female behaviour during agonistic encounters. Both sexes responded to territorial intrusions by displaying confrontational and non-confrontational behaviour. A recent study by Velasco, Ferrer and Sanz [34] using clay decoys placed half a meter from the nest box, described three non-mutually exclusive strategies used by male blue tits during male–male territorial conflicts which could be ranked from high to low risk: a confrontational, a non-confrontational intimidating and a non-confrontational cautious strategy. In contrast, our principal component analysis did not reveal clearly distinguishable strategies, although we found similar high- and low-risk behaviours. Some individuals scored high across multiple components indicating behaviours associated with the components are not mutually exclusive. We consider the first component for both sexes as a reliable proxy of confrontational aggressive behaviour in the context of territorial intrusions, as this mainly included direct physical interactions with the decoy. Non-confrontational behaviour varied between both sexes. Non-confrontational females either called from a distance or entered the nest box more frequently and for longer periods of time, while some females both called and entered the nest box frequently. Females may call to signal their partner or to deter intruders without physical contact [40]. The importance of entering the nest box during territorial intrusions is unclear. Females likely do so to check on or protect the nest, or to hide from the intruder. Non-confrontational males either sang at a distance, or called. Females did not sing during our intrusion experiments, although female song is common in the blue tit [44]. Female song may be a long-distance signal, and less useful at deterring intruders in close proximity. However, Sierro et al. [44] reported females both attacking and singing during agonistic interactions. Moreover, other studies, on the superb fairy-wren (*Malurus cyaneus*), eastern whipbirds (*Psophodes olivaceus*), and bluethroat (*Luscinia svecica*), showed that females do respond with song to simulated territorial intrusions [13,45,46]. However, these studies utilised playback, which may trigger an alternative behavioural response. Finally, it should be noted that for both sexes, the components describing non-confrontational behaviour should be interpreted with caution, as they were largely determined by only a few individuals. In addition, in our analysis, we did not take into account whether males or females were first to arrive and if partners were present, although the behaviour of an individual may be influenced by the presence of their partner. Our results show that female and male blue tits largely use similar behaviours during agonistic encounters, although there are sex-specific differences with regard to the expression of song and attention directed towards the nest. This difference may be the result of the higher cost of losing a nest site during egg-laying for females compared to males, resulting in females taking more risks by entering the nest box and males being more cautious by displaying signalling aggression.

In contrast to previous studies [3,4,9,11,12,13,17], including research on the closely related great tit [4], we did not find evidence that female blue tits responded differently to same-sex and opposite-sex intruders (i.e., sex-specific differences in female aggression). By contrast, male blue tits showed greater non-confrontational aggressiveness towards male intruders than female intruders, as measured by one of the three PCs (PC2). Males confronted with a male decoy sang more at a distance than in presence of a female decoy, but direct confrontational aggression did not differ. Male confrontational aggression towards same-sex intruders was expected to be higher to ensure the paternity of the brood. A previous study has shown that aggression towards same- and opposite-sex intruders varies across species, though this may depend on breeding stage [11]. Males may be particularly aggressive toward same-sex intruders early in the breeding season when territories are undefined and when females are fertile [11]. However, during the egg-laying stage, males have invested less in the brood than females, and may therefore be less willing to risk a direct confrontation. As the breeding season progresses, males may become more aggressive as the cost of losing a nest increases for the male, although a study on western bluebirds (*Sialia mexicana*) did not find differences in male aggression across the egg-laying and incubation stage [47]. The absence of sex-specific aggression in female blue tits might be related to the strong competition for nesting sites both with blue tits and great tits and/or their short life expectancy. In many blue tit populations including ours, great tits dominate blue tits in direct competition for nest sites [35,36]. Moreover, many blue tit females breed only once during their lifetime. In accordance with the pace-of-life syndrome hypothesis, this may drive selection for aggressive phenotypes, regardless of the sex of the intruder, to ensure the survival of a female’s current brood [48]. The fact that males in this population mainly displayed song during encounters with male decoys, suggests it has an intrasexual function. However, this result should be interpreted with caution, as in total only five males produced song during territorial intrusions, and when considering aggression variables separately, no sex-specific differences were identified. Further research across different breeding stages is necessary to better understand why intruder sex affects male behaviour but not female behaviour.

The relative levels of confrontational aggression (PC1) during intra- and intersexual conflicts were consistent for females, but not males. This suggests that, in females, same- and opposite-sex aggression may not be independent and may be regulated by common physiological mechanisms. Alternatively, individuals may assess stimuli from the intruder and regulate their response in a similar manner during both intra- and intersexual conflicts [3]. Our results contradict previous research on mice, where female aggression is regulated by different neurochemical mechanisms and motivations based on the sex of the intruder [30]. Furthermore, while highly aggressive male mice respond to intruders of both sexes, less aggressive male mice only attack male intruders [29]. However, Cain et al. [3], using live caged conspecifics placed three meters from the nest, showed that female dark-eyed juncos (*Junco hyemalis carolinensis*) are consistent in the level of aggression towards same- and opposite-sex intruders. Why female, but not male birds are consistent in their confrontational aggression remains unclear. If intra-and intersexual aggression are mediated by the same mechanisms, they might be affected by the same selection pressures and not evolve independently. Further research is needed to determine what drives this consistency in females but not males. 

Female blue tits displayed a higher intensity of aggression than males, approaching and perching on the decoy more frequently and spending more time on the decoy and in the nest box, regardless of the sex of the intruder. Additionally, although female aggression remained consistent overall, there were subtle variations in the presence of a female intruder, such as increased pecking and time spent in the nest-box entrance. These behaviours demonstrate a high level of female aggression, which is to be expected in a species where competition for nesting sites is high and incidents of takeovers and brood parasitism occur [49]. Previous research has indeed shown that territorial aggression is crucial for obligate cavity-nesting species to gain and maintain access to suitable nesting sites [1,31,49,50]. Similar to blue tits, female tree swallows, a cavity-nesting species with a strong competition for nest holes, attack intruders (taxidermic mounts placed on the nest box) at a higher rate compared to males [11]. This suggests that females of cavity-nesting species are indeed more aggressive due to strong nest-site competition. Females often face greater reproductive consequences if the nest is destroyed than males and their reproductive success is almost completely dependent on access to a nest site [49,51]. As previously mentioned, during the egg-laying phase, females have invested more than males in nest development and maintenance. Consequently, trade-offs between the cost of losing the nest and risking injury may differ between the sexes, explaining male behaviour. In contrast to females, male blue tits were more often cautious, stayed at a distance, called more, approached the decoy less, and never entered the nest box. Due to the disparity of investments, males may be inclined to use indirect means of confrontation, such as singing at a distance to intimidate intruders, rather than risking a direct physical confrontation. Alternatively, females may be protecting the nest itself, while males protect the wider territory. Previous research showed that great tits defend empty nest boxes against live heterospecific intruders up to at least 25 m from their own nest box [52]. Our experimental setup with an intruder directly on top of the nest box might have elicited a stronger female response, as they have invested more in the nest [38]. However, Lipshutz and Rosvall [11], similar to our study, used taxidermic mounts placed on top of the nest box, or within 0.5 m of the nest, and found that only one out of the four study species showed a difference in the intensity of the responses between sexes. Nevertheless, it remains interesting to examine how both male and female behaviour may change as the decoy is placed further from the nest box. Moreover, the response of males and females may be dependent on the age of the decoys, although our results indicate that the response did not depend on which decoy was used. Finally, using a taxidermy mount as decoy precludes the possibility of aggressive behaviour from the intruder. In contrast to our results, a previous study using live caged intruders showed that male blue tits respond strongly to intruders near the nest box [53]. Blue tits may react differently to a live decoy, which could decrease the response of females due to increased perceived risk, or incite more aggressive nest defence from males. Despite these potential limitations, our results suggest that females may be the more aggressive sex during simulated territorial intrusions during egg-laying in the blue tit. However, clearly, future studies should address whether our results also hold when studying other breeding stages than egg laying or other populations (including rural/forest populations) and when decoys are placed further away from the nest box or when live intruders/decoys are used. 

A small number of both male and female blue tits directed aggression towards their partner. Resident females showed aggression towards their partner during both same- and opposite-sex intruder conflicts, although this might have been in reaction to the aggression from the resident male. Only the most aggressive males showed within-pair aggression, and exclusively in the presence of a male intruder. In other species, such as red-winged blackbirds and European starlings, male within-pair aggression has been reported as a means to increase their chances of becoming polygynous, by deterring female aggression on secondary females [22,23]. Male blue tits likely use within-pair aggression to ensure paternity by preventing extra pair copulations of their partner. Gowaty [17] reported that male Eastern bluebirds (*Sialia sialis*) directed aggression towards their partners in the presence of a male intruder. Additionally, similar to our study, they found a significant positive correlation between male aggression towards a male decoy and towards their mate. Our results suggest that blue tit males use within-pair aggression as a form of mate guarding, preventing the female from approaching an intruding male. Further research is needed to confirm this hypothesis, and determine if individuals showing within-pair aggression are more successful at preventing extra-pair copulations.

## 5. Conclusions

Female aggression has long been understudied. Here, we provide evidence that females may be the more aggressive sex during simulated territorial intrusions during the egg-laying period in blue tits. Although both sexes display similar confrontational behaviours during such intrusions, females were never observed singing, and males never entered the nest box. Females may be more aggressive as a result of the varying costs associated with losing the nest for each sex. Specifically, high nest-site competition may be an important selective pressure that drives female aggression. While aggression was mostly independent of the sex of the intruder, males sang more from a distance during male–male encounters. Males may opt for a low-risk nest defence strategy during the egg-laying period, as their investment is lower than that of females. Consequently, male confrontational aggressive behaviour may increase throughout the breeding stages. Individual levels of female, but not male, confrontational aggression were consistent during intra- and inter-sexual intrusions. Future studies should examine whether sex differences persist across breeding stadia, as well as focus on the causes and consequences of individual and sex differences in aggressive behaviour.

## Figures and Tables

**Table 1:** Results from the principal component analysis for females, including eigenvalues, percentage of variance explained and factor loadings. Data include responses on both male (*n* = 26) and female (*n* = 26) decoys. The minimum distance is reversed (multiplied by −1), so larger values indicate more confrontational aggression. The factor loadings for PC1 are reversed (multiplied by −1) for the same reason. Variables with loadings greater than 0.40, deemed important for the construction of the components, are highlighted in bold.

Female (*n* = 26)			
	PC1	PC2	PC3
Eigenvalue	3.24	1.98	1.02
Variance %	40.53	24.76	12.80
**Variable**	**Loadings**	**Loadings**	**Loadings**
Pecks	**0.45**	0.064	0.18
Time on decoy	**0.50**	0.087	−0.18
Calls	−0.072	**0.40**	**0.59**
Minimum distance	0.30	−0.34	**−0.47**
Time in entrance	**0.41**	0.037	0.30
Time in nest box	−0.14	**−0.62**	0.24
Decoy frequency	**0.50**	0.052	0.092
Nest-box frequency	0.12	**−0.57**	**0.46**

**Table 2:** Results from the principal component analysis for males, including eigenvalues, percentage of variance explained, and factor loadings. Data include responses on both male (*n* = 24) and female (*n* = 24) decoys. The minimum distance is reversed (multiplied by −1), so larger values indicate more confrontational aggression. Similarly, the factor loadings for PC1 are reversed (multiplied by −1) for the same reason. Variables with loadings greater than 0.40, deemed important for the construction of the components, are highlighted in bold.

Male (*n* = 24)			
	PC1	PC2	PC3
Eigenvalue	2.60	1.11	1.06
Variance %	43.25	18.55	17.74
**Variable**	**Loadings**	**Loadings**	**Loadings**
Pecks	**0.50**	0.35	0.14
Time on decoy	**0.57**	0.0035	0.015
Calls	−0.12	0.19	**0.88**
Song	−0.15	**0.73**	**−0.42**
Minimum distance	**0.41**	**−0.46**	0.15
Time in entrance	**0.48**	0.31	0.048

## Data Availability

The data presented in this study are available in the Supplementary Materials: Data S1.

## Supplementary Materials

The following supporting information can be downloaded at: https://www.mdpi.com/article/10.3390/ani13040585/s1, Data S1: Simulated territorial intrusions.

## Appendix A

**Table A1:** Descriptive statistics of the aggression parameters scored during simulated territorial intrusions for both sexes and per decoy (N_Female_ = 26; N_Male_ = 24). Mean, standard deviation (SD), minimum (min), median and maximum (max) values are given. ‘Prop’ indicates the proportion of individuals which perform the behaviour during the simulated territorial intrusions. For the parameters ‘Time on decoy (s)’, ‘Time in entrance (s)’ and ‘Time in nest box (s)’, ‘prop’ refers to the proportion of tests in which individuals spent at least one second displaying the behaviour. For ‘Minimum distance (m)’, ‘prop’ indicates the proportion of individuals which perched on the decoy.

		Female Decoy					Male Decoy				
Variable ^†^	Sex	Mean ± SD	Min	Median	Max	Prop	Mean ± SD	Min	Median	Max	Prop
Pecks	Female	17.58 ± 22.46	0	9	82	73.08	6.81 ± 7.47	0	5.5	24	73.08
	Male	0.25 ± 0.68	0	0	3	16.67	12.54 ± 29.66	0	0	102	20.83
Time on decoy (s)	Female	101.7 ± 75.25	0	91.5	226	96.15	84.15 ± 71.23	0	67.5	226	92.31
	Male	19.08 ± 28.04	0	3.5	100	54.17	39.17 ± 71.87	0	0	218	41.67
Calls	Female	1.65 ± 6.49	0	0	33	19.23	1.62 ± 5.73	0	0	29	23.08
	Male	22.54 ± 25.18	0	14.5	90	75.00	24.79	0	23	61	83.33
Song	Female	0 ± 0	0	0	0	0.00	0 ± 0	0	0	0	0.00
	Male	0.04 ± 0.20	0	0	1	4.17	2.88	0	0	51	16.67
Minimum distance (m)	Female	0.08 ± 0.39	0	0	2	96.15	0.08 ± 0.39	0	0	2	92.31
	Male	0.72 ± 1.18	0	0.05	4	54.17	1.67	0	0.2	15	41.67
Time in entrance (s)	Female	14.5 ± 23.23	0	1	98	50.00	14.73 ± 19.98	0	6.5	79	53.85
	Male	7.83 ± 32.84	0	0	160	12.5	10.33	0	0	126	20.83
Time in nest box (s)	Female	91.31 ± 110.44	0	50	297	61.54	107.4 ± 109.63	0	78.5	275	69.23
	Male	0 ± 0	0	0	0	0.00	0	0	0	0	0.00
# on decoy	Female	11.12 ± 10.48	0	9	42	96.15	9.58 ± 10.87	0	6.5	44	92.31
	Male	3 ± 3.91	0	1	11	54.17	3.71	0	0	25	41.67
# in nest box	Female	1.65 ± 2.58	0	1	12	61.54	1.5 ± 1.86	0	1	6	65.38
	Male	0 ± 0	0	0	0	0.00	0	0	0	0	0.00

^†^ Variables: The number of pecks (Pecks), time on the decoy (Time on decoy), number of calls (Calls), number of song phrases (Song), minimum distance to the decoy (Minimum distance), time in front of the nest-box entrance (Time in entrance), the time in the nest box (Time nest box), the number of times perching on the decoy (# on decoy) and the number of times entering the nest box (# in nest box).

**Table A2:** Results of Wilcoxon signed rank tests comparing aggression variables for both sexes (N_Female_ = 26; N_Male_ = 24) between the trials on female and male decoys. The median, test statistic and adjusted *p*-values with the Holm correction for multiple testing are presented [43].

		Decoy		Wilcoxon Signed Rank Test (Paired)	
		Female (N = 26)	Male (N = 24)		
Variable ^†^	Sex	Median	Median	V	Adjusted *p*-Value
Pecks	Female	9	5.5	203	0.21
	Male	0	0	10	1.0
Time on decoy	Female	91.5	67.5	217	1.0
	Male	3.5	0	47	1.0
Calls	Female	0	0	30.5	1.0
	Male	14.5	23	96.5	1.0
Song	Female	/	/	/	/
	Male	0	0	1.5	0.95
Minimum distance	Female	0	0	2.5	1.0
	Male	0.05	0.2	54.5	1.0
Time in entrance	Female	1	6.5	69.5	1.0
	Male	0	0	6	1.0
Time in nest box	Female	50	78.5	85	1.0
	Male	/	/	/	/
# on decoy	Female	9	6.5	217.5	1.0
	Male	1	0	66.5	1.0
# in nest box	Female	1	1	74.5	1.0
	Male	/	/	/	/

^†^ Variables: The number of pecks (Pecks), time on the decoy (Time on decoy), number of calls (Calls), number of song phrases (Song), minimum distance to the decoy (Minimum distance), time in front of the nest-box entrance (Time in entrance), the time in the nest box (Time nest box), the number of times perching on the decoy (# on decoy) and the number of times entering the nest box (# in nest box).

**Table A3:** Results of Mann–Whitney U tests comparing how female and male blue tits (N_Female_ = 26; N_Male_ = 24) responded to same-sex and opposite-sex rivals for the different behaviours considered. The median, test statistic and adjusted *p*-values with the Holm correction for multiple testing are presented [43]. Asterisks indicate level of statistical significance: * *p* ≤ 0.05, ** *p* ≤ 0.01, *** *p* ≤ 0.001.

		Sex		Mann–Whitney U Test		
		Female (N = 26)	Male (N = 24)			
Variable ^†^	Decoy	Median	Median	U	Adjusted *p*-Value	
Pecks	Female	9	0	525	1.0 × 10^−4^	***
	Male	5.5	0	428	0.064	
Time on decoy	Female	91.5	3.5	525.5	3.4 × 10^−4^	***
	Male	67.5	0	479.5	0.0059	**
Calls	Female	0	14.5	108.5	2.1 × 10^−4^	***
	Male	0	23	82	3.9 × 10^−5^	***
Song	Female	0	0	299	0.32	
	Male	0	0	260	0.10	
Minimum distance	Female	0	0.05	171	0.0034	**
	Male	0	0.2	149	0.0014	**
Time in entrance	Female	1	0	427.5	0.035	*
	Male	6.5	0	408	0.10	
Time in nest box	Female	50	0	504	1.0 × 10^−4^	***
	Male	78.5	0	528	2.0 × 10^−5^	***
# on decoy	Female	9	1	494	0.0034	**
	Male	6	0	483.5	0.0050	**
# in nest box	Female	1	0	504	1.0 × 10^−4^	***
	Male	1	0	516	4.0 × 10^−5^	***

^†^ Variables: The number of pecks (Pecks), time on the decoy (Time on decoy), number of calls (Calls), number of song phrases (Song), minimum distance to the decoy (Minimum distance), time in front of the nest-box entrance (Time in entrance), the time in the nest box (Time nest box), the number of times perching on the decoy (# on decoy) and the number of times entering the nest box (# in nest box).
